# Narrow band imaging versus white light for detecting sessile serrated lesion: A prospective randomized multicenter study

**DOI:** 10.1002/deo2.44

**Published:** 2021-09-01

**Authors:** Dominic Staudenmann, Ken Liu, Poornima Varma, May Wong, Sonam Rai, Tatiana Tsoutsman, Kyung Ho Choi, Payal Saxena, Arthur John Kaffes

**Affiliations:** ^1^ AW Morrow Gastroenterology and Liver Centre Royal Prince Alfred Hospital Sydney Australia; ^2^ Department of Gastroenterology Praxis Balsiger Seibold und Partner Bern Switzerland; ^3^ Université de Fribourg Fribourg Switzerland; ^4^ Sydney Medical School University of Sydney Sydney Australia; ^5^ Department of Gastroenterology Austin Health Heidelberg Australia; ^6^ Royal North Shore Hospital St. Leonards Sydney Australia

**Keywords:** adenoma detection rate, colonoscopy, narrow band imaging, polypectomy, sessile serrated lesion

## Abstract

**Objectives:**

Colonoscopy is the gold standard diagnostic test used to detect early colorectal lesions and prevent colorectal carcinoma. Narrow band imaging (NBI) is an imaging technique that provides improved image resolution of the mucosa during endoscopy. Whether NBI improves the detection of sessile serrated lesion (SSL) is controversial—our aim was to assess this during routine colonoscopy.

**Methods:**

We conducted a multicenter, prospective, randomized, controlled trial. Patients underwent colonoscopy for screening, surveillance, or symptoms. They were randomized to either high‐definition white light (HD‐WL) or NBI in a 1:1 ratio. The primary outcome was SSL detection rate. Secondary outcomes were adenoma detection rate (ADR) and polyp detection rate (PDR).

**Results:**

A total of 400 patients were randomized to NBI (N = 200) or HD‐WL (N = 200). The total colonoscopy time was slightly longer in the NBI group compared to HD‐WL (median time 14 vs. 12 min, *p* = 0.033). There were no statistically significant differences in SSL detection rate (7.5% NBI vs. 8.0% HD‐WL; *p* = 0.852), ADR (41.0% NBI vs. 37.5% HD‐WL; *p* = 0.531), or PDR (61.0% NBI vs. 54.0% HD‐WL; *p* = 0.157) between the two groups. No significant predictors of SSL detection were found on univariable or multivariable analysis. Increasing age and increased withdrawal time were an independent predictors of polyp detection and increasing age was also an independent predictor of adenoma detection on multivariable analysis.

**Conclusion:**

In the hands of experienced colonoscopists, NBI does not improve SSL detection compared to HD‐WL. Withdrawal time and patient age remain important factors for polyp and adenoma detection.

**Abbreviations**
ADR: adenoma detection rate
aOR: adjusted OR
BBPS: Boston Bowel Preparation Scale
CI: 95% confidence intervals
CRC: colorectal carcinoma and with
HD‐WL: high‐definition white light endoscopy
IQR: interquartile range
NBI: Narrow band imaging
OR: odds ratios
PDR: polyp detection rate
SSL: sessile serrated lesion
SSLDR: SSL detection rate
VIF: Variance inflation factor

## INTRODUCTION

Colorectal carcinoma (CRC) is the second most common cause of cancer‐related death in Australia and the world.^1^ Although colonoscopy with identification and removal of precursor lesions is considered the most effective method at preventing CRC, it is an imperfect tool with adenoma miss rates ranging from 15 to 27%.^2^, ^3^ This is especially true for sessile serrated lesions (SSLs), as these lesions are often flat, inconspicuous, and have an overlying mucous cap.^3^, ^4^ In addition, SSLs are frequently found in the right colon, where bowel preparation tends to be poorer. Moreover, due to their indistinct borders, they are often incompletely resected and are thus associated with interval and synchronous CRC.^3^, ^5^, ^6^ It is estimated that up to 30% of all CRCs emerge from SSLs via the serrated neoplasia pathway.^7^ As a result, it is important that SSLs are correctly identified and completely resected when they are encountered during colonoscopy. Different methods and techniques have been used to improve the SSL detection rate (SSLDR) with minimal success. Fecal immunochemical test has been shown to be poorly sensitive for detecting SSLs including large ones.^8^ Ancillary devices used during colonoscopy have also failed to demonstrate improved SSL detection.^9^, ^10^ There is some evidence that chromoendoscopy might improve SSLDR compared to standard white light.^11^, ^12^

Narrow band imaging (NBI) is a technique of image enhancement that alters white light into narrowed bands of light with a center wavelength of 415 nm (blue) and 540 nm (green) which enhances the visualization of blood vessels and mucosal pit patterns.^13^ This results in increased contrast between adenomas and adjacent normal colonic mucosa because adenomas are more vascular.^14^ A recent study suggested NBI may increase the detection of proximal colon serrated lesions but this result did not reach statistical significance.^15^ The authors concluded that additional study on the use of NBI for SSL detection was warranted. Therefore, we performed a prospective randomized study to evaluate whether NBI improves the detection of SSLs during routine colonoscopy.

## MATERIALS AND METHODS

### Study design and patients

This study was a prospective, randomized controlled study involving three university teaching hospitals across Australia (registration number ACTRN12616000395437p) and designed as a superiority trial. The study was conducted according to the NHMRC National Statement on Ethical Conduct in Human Research (2007) and all its updates^16^ and was approved by the Sydney Local Health District Human Ethics Research Committee (RPAH Zone), reference X16‐0338 & HREC/16/RPAH/464.

**FIGURE 1:**
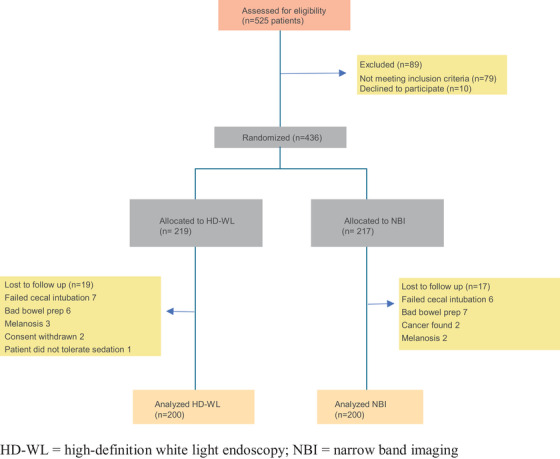
Patient flow. HD‐WL, high‐definition white light endoscopy; NBI, narrow band imaging

Consecutive patients referred for colonoscopy for screening, surveillance, or colonic symptoms during the study period between October 2017 and November 2020 were invited to participate. Written informed consent was obtained from all recruited subjects. Inclusion criteria were patients over the age of 18 who were able to give informed consent. Exclusion criteria were history of inflammatory bowel disease, acute upper gastrointestinal hemorrhage, anticoagulation by any drug other than aspirin, and standard contraindications to colonoscopy or sedation. Subjects were also excluded after enrollment for failure to reach the cecum, presence of melanosis coli, consent withdrawal, diagnosis of CRC, intolerance to sedation and/or inadequate bowel preparation (Figure 1).

### Randomization

Patients were randomized to each modality in a 1:1 ratio in blocks of 10 by a computer‐generated sequence. Randomization was revealed at the beginning of the procedure.

### Colonoscopy

All subjects received dietary instructions before colonoscopy and polyethylene glycol in a split dose for bowel preparation one day prior to the colonoscopy. Patients were consciously sedated using fentanyl, midazolam and/or propofol. The procedures were conducted by consultant gastroenterologists and fellows in gastroenterology who have been accredited by the Conjoint Committee for the Recognition of Training in Gastrointestinal Endoscopy. The two fellows participating in the study had at least 5 years of experience and more than 3000 colonoscopies performed. Each endoscopist performed colonoscopies roughly equally in both groups and the background of the endoscopists was similar in both groups. Prior to study commencement, all endoscopists received a standardized 60 min training module reviewing the study protocol, methods, and data recording. Colonoscopic technique was standardized and included: reaching the cecum, attempted retroflexion in the cecum (in order to gain a second look at the cecum and the ascending colon), attempted retroflexion in the rectum and a double look at all the flexures (splenic and hepatic) and the sigmoid colon, and a minimum of 6 min spent withdrawing the colonoscope. No endoscopic accessories, for example, endoscopic caps were used.

All colonoscopes were performed with high‐definition white light endoscopy (HD‐WL) until the cecum was reached, and bowel preparation quality had been assessed. The quality of the bowel preparation was assessed according to the Boston Bowel Preparation Scale (BBPS) which assesses the ascending, transverse, and descending colon on a scale of 0 to 3 for a total score out of 9.^17^ Colonoscopies were performed with the 190‐series Olympus colonoscope which is capable of switching between NBI and HD‐WL. The entire withdrawal phase was performed with the assigned imaging type modality and the withdrawal time was measured (time for polypectomy was not included).

### Polyps

All polyps detected during colonoscopy were documented for their location, morphology, and size. All polyps apart from multiple hyperplastic rectal polyps were removed and sent to a pathologist blinded to the assigned modality for histopathological characterization. The WHO 2010 pathological criteria were used.^18^ After each polypectomy, the colonoscope was switched back to the assigned modality if it had been changed for the polypectomy so withdrawal could continue under the assigned modality.

Polyps were defined histologically as:

- Adenomas including tubular, villous, and tubulovillous adenomas.
- SSL encompassing sessile serrated adenomas or polyps and traditional serrated adenomas.
- Hyperplastic polyps.

Histologically SSLs were distinguished from conventional hyperplastic polyps on the basis of crypt dilation, irregularly branching crypts, and horizontally arranged basal area of the crypts. Moreover, Ki‐67 immunostaining was used as another diagnostic tool in order to differentiate SSL from hyperplastic polyps. The number and distribution of Ki67‐positive cells differed between hyperplastic polyps and SSL. The latter have a higher Ki67‐positive rate and an asymmetrical distribution.^19^, ^20^

### Sample size

SSLDRs using HD‐WL have been reported to be between 2 and 8.2% in previous studies.^21^, ^22^, ^23^, ^24^ At time of study design, very few studies have specifically examined SSLDR using NBI. Therefore, for our sample size calculation, we hypothesized the SSLDR to be at 2% for the HD‐WL group (the lower end of published SSLDR for HD‐WL) and 8.2% for the NBI group (the upper end of published SSLDR for HD‐WL). With a two‐sided significance level of 5%, and 80% power, we estimated that a sample size of 392 patients (196 in each arm) was necessary to detect the indicated difference. To account for patients who may be excluded after enrolment (e.g., due to poor bowel preparation), we recruited an extra 10% in each arm (i.e., total of at least 216 patients in each arm).

### Statistical analysis

Statistical analysis was performed by Statistical Package for Social Science (SPSS version 22.0, Armonk, NY, USA). Continuous variables were expressed in mean ± standard deviation or median (interquartile range [IQR]) as appropriate. Differences between subgroups were analyzed using *χ*2 or Fisher's exact test for categorical parameters and Student's *t*‐test or Mann–Whitney test for continuous parameters as appropriate. The primary outcome of interest was SSLDR with secondary outcomes of polyp detection rate (PDR) and Adenoma detection rate (ADR). Multivariable binary logistic regression model using backward stepwise selection based on likelihood ratio test was performed on predictors with *P *< 0.20 in univariable analysis to determine independent factors associated with polyp detection, adenoma detection, and SSL detection. Variance inflation factor (VIF) was used to detect multicollinearity between covariates with a VIF >5 considered as significant multicollinearity. Odds ratios (OR) and adjusted OR (aOR) with 95% confidence intervals (CI) of the predictors were computed. All statistical tests were two‐sided. Statistical significance was taken as *p *< 0.05.

## RESULTS

### Patient demographics

A total of 436 eligible patients underwent colonoscopy during the study period. Of these, 36 drop out cases (8.3%) were excluded from the study after completion of colonoscopy: 17 (3.9%) in the NBI arm and 19 (4.4%) in the HD‐WL arm. The remaining 400 patients (200 in the HD‐WL arm and 200 in the NBI arm) completed the study protocol (Figure 1). There were equal numbers of males and females (50%). The median age was 60 years (IQR: 49‐69). Surveillance due to previous polyps was the most common indication for a colonoscopy (30.0%). There was a slightly higher proportion of patients having colonoscopy for the indication of family history of CRC in the HD‐WL group compared to NBI (18.0 vs. 11.0%, *p* = 0.047) (Table 1). The median Boston bowel preparation score was an 8 out of 9 (IQR 6–9). There was also a significant difference between the total colonoscopy time with NBI taking longer than HD‐WL (14 min [IQR 11‐17] vs. 12 min [IQR 12‐16], *p* = 0.033) and a very small but statistically significant difference in median withdrawal time between the two groups (7 min [IQR 7‐9] for NBI vs. 7 min [IQR 6‐8] for HD‐WL, *p *< 0.001). Otherwise, there were no significant differences between NBI and HD‐WL groups (Table 2).

**TABLE 1:** Baseline characteristics of patients

Characteristic	NBI	HD‐WL	***p***
Male sex (%)	51.5	48.5	0.549
Median age (IQR)	61 (59‐70)	59 (49‐69)	0.369
Prior colonoscopy (%)			
None	39.0	34.5	0.913
<1 year	5.0	5.0	
1‐3 years	17.0	17.5	
3‐5 years	15.0	17.0	
>5 years	24.0	26.0	
Previous polyps (%)	50.4	47.4	0.628
Previous CRC (%)	1.5	0.5	0.315
Anticoagulants or antiplatelets (%)	9.3	4.5	0.094
Indication for colonoscopy (%)			
Previous polyps	31.0	29.0	0.663
**Family history of CRC**	**11.0**	**18.0**	**0.047**
Overt PR bleeding	12.5	16.0	0.317
Positive FOBT	14.0	11.5	0.454
Altered bowel habit	12.5	10.5	0.531
Abdominal pain	9.5	5.0	0.083
Family history of polyps	4.5	5.0	0.814

CRC, colorectal cancer; FOBT, fecal occult blood test; HD‐WL, high‐definition white light endoscopy; IQR, interquartile range; NBI, narrow band imaging.

**TABLE 2:** Polyp, adenoma, and SSL detection rates in NBI and HD‐WL groups

Characteristic	NBI	HD‐WL	*p*
Median BBPS (IQR)	8 (6‐9)	8 (6‐9)	0.563
Median time (IQR)			
To cecum	5 (4‐9)	6 (4‐10)	0.958
**Withdrawal**	7 (7‐9)	7 (6‐8)	**<0.001**
**Total**	14 (11‐17)	12 (10‐16)	**0.033**
Polyp detection rate (%)	61.0	54.0	0.157
Median number of polyps detected	1 (0‐2)	1 (0‐2)	0.241
Median polyp max size (mm)	5 (3‐6)	5 (4‐8)	0.188
Adenoma detection rate (%)	37.0	34.0	0.531
Median number of adenomas detected	0 (0‐0)	0 (0‐1)	0.426
SSL detection rate (%)	7.5	8.0	0.852
Median number of SSL detected	0 (0‐1)	0 (0‐0)	0.859
Median polyps per patient with polyp	1 (1‐3)	2 (1‐3)	0.801
Median adenomas per patient with adenoma	1 (1‐2)	1 (1‐3)	0.403
Median SSL per patient with SSL	1 (1‐2)	1 (1‐2)	0.824

BBPS, Boston Bowel Preparation Scale; HD‐WL, high‐definition white light endoscopy; IQR, interquartile range; NBI, narrow band imaging; SSL, sessile serrated lesion.

### SSL, polyp, and adenoma detection rates

The overall SSLDR was 7.8% with no statistically significant difference between NBI v HD‐WL (7.5 vs. 8.0%, respectively, *p* = 0.852). There was also no statistically significant difference in the median (1 [IQR 1‐2] vs. 1 [IQR 1‐2], *p* = 0.824) of SSLs detected per patient with SSL. The overall PDR was 57.5% and the ADR was 35.5%. There were also no statistically significant differences between the two arms for these outcomes (PDR: NBI 61.0% vs. HD‐WL 54.0%, *p* = 0.157; and ADR: NBI 37.0% vs. HD‐WL 34.0%, *p* = 0.531). The unadjusted OR for NBI use were: 1.932, 95%CI 0.448‐1.941, *p* = 0.852 for SSLDR; 1.332, 95%CI 0.895‐1.983, *p* = 0.157 for PDR; and 1.140 95%CI 0.757‐1.718, *p* = 0.531 for ADR. Table 2 summarizes the SSLDR, PDR, and ADR in each study arm.

### Predictors of SSL, polyp, and adenoma detection

There were no significant predictors of SSL detection on univariable analysis (Table 3). Increased age (OR 1.039 per year increase, 95%CI 1.020‐1.059, *p *< 0.001) and withdrawal time (OR 1.129 per min increase, 95% CI 1.002‐1.272, *p* = 0.046) were independently associated with polyp detection multivariable analysis. Only increased age was an independent predictor of adenoma detection (OR 1.038 per year increase, 95% CI 1.018‐1.057, *p *< 0.001). There was no significant collinearity between the covariates analyzed in the multivariable analysis for both polyp and adenoma detection (VIF <2 for all). Use of NBI instead of HD‐WL was not associated with SSL, polyp or adenoma detection on univariable or multivariable analysis. Univariable and multivariable predictors of polyp and adenoma detection are listed in Table 4.

**TABLE 3:** Univariable predictors of SSL detection

	Univariate		
Variable	OR	95%CI	*p*
NBI vs. HD‐WL	0.932	0.448‐1.941	0.852
Age (per year increase)	0.993	0.968‐1.019	0.615
Sex (male vs. female)	0.608	0.287‐1.289	0.194
Prior colonoscopy (Y vs. N)	1.477	0.657‐3.318	0.345
Anticoagulant/antiplatelet (Y vs. N)	1.310	0.286‐5.986	0.728
Indications for colonoscopy			
Rectal bleeding (Y vs. N)	1.172	0.431‐3.190	0.756
Abdominal pain (Y vs. N)	0.406	0.053‐3.089	0.384
Altered bowel habit (Y vs. N)	0.509	0.117‐2.209	0.368
Positive FOBT (Y vs. N)	1.015	0.340‐3.030	0.979
Family history of CRC (Y vs. N)	1.147	0.422‐3.118	0.789
Previous polyps (Y vs. N)	1.312	0.608‐2.831	0.489
Family history of polyps (Y vs. N)	1.428	0.314‐6.485	0.644
Time taken to reach cecum (per minute increase)	0.990	0.908‐1.078	0.815
Withdrawal time (per minute increase)	0.996	0.838‐1.185	0.966
Total colonoscopy time (per minute increase)	1.003	0.929‐1.083	0.943
BBPS (per point increase)	1.006	0808‐1.251	0.959
Retroflexion in the rectum (Y vs. N)	0.729	0.479‐1.109	0.140

BBPS, Boston Bowel Preparation Scale; CI, confidence interval; CRC, colorectal cancer; FOBT, fecal occult blood test; HD‐WL, high‐definition white light endoscopy; NBI, narrow.

**TABLE 4:** Univariable and multivariable predictors of polyp detection and adenoma detection

	Univariate			Multivariate		
Variable	OR	95%CI	*p*	aOR	95% CI	*p*
PREDICTORS OF POLYP DETECTION						
NBI vs. HD‐WL	1.332	0.895‐1.983	0.157	1.132	0.662‐1.936	0.651
Age (per year increase)	**1.041**	**1.025‐1.057**	**<0.001**	**1.039**	**1.020‐1.059**	**<0.001**
Prior colonoscopy (Y vs. N)	1.467	0.968‐2.221	0.071	0.720	0.394‐1.317	0.286
Anticoagulant/antiplatelet (Y vs. N)	1.949	0.742‐5.123	0.176	1.066	0.371‐3.064	0.905
Abdominal pain indication (Y vs. N)	0.424	0.195‐0.924	0.031	0.795	0.302‐2.091	0.641
Altered bowel habit indication (Y vs. N)	0.583	0.314‐1.081	0.087	0.830	0.363‐1.896	0.658
Previous polyp indication (Y vs. N)	2.142	1.358‐3.377	0.001	1.674	0.882‐3.178	0.115
Withdrawal time (per minute increase)	**1.115**	**1.009‐1.234**	**0.033**	**1.129**	**1.002‐1.272**	**0.046**
Retroflexion in the rectum (Y vs. N)	0.741	0.496‐1.106	0.142	0.705	0.402‐1.238	0.224
PREDICTORS OF ADENOMA DETECTION						
Age (per year increase)	**1.045**	**1.028‐1.063**	**<0.001**	**1.038**	**1.018‐1.057**	**<0.001**
Anticoagulant/antiplatelet (Y vs. N)	3.183	1.293‐7.835	0.012	2.214	0.877‐5.589	0.093
Abdominal pain indication (Y vs. N)	0.556	0.232‐1.336	0.190	0.948	0.358‐2.507	0.914
Previous polyps indication (Y vs. N)	1.877	1.210‐2.913	0.005	1.150	0.690‐1.916	0.592
Retroflexion Rectum (Y vs. N)	0.729	0.479‐1.109	0.140	0.753	0.467‐1.213	0.243

aOR, adjusted odds ratio; CI, confidence interval; HD‐WL, high‐definition white light endoscopy; NBI, narrow band imaging; OR, odds ratio.

## DISCUSSION

Endoscopic detection of SSLs is notoriously challenging.^3^, ^4^ Wide variability in reported SSLDR has been reported due to factors related to the patient, the endoscopists, and the procedure.^25^, ^26^ Our SSLDR was 7.8%, which is comparable to the most recent studies.^24^, ^27^, ^28^ In this prospective, randomized controlled trial, NBI was not found to be significantly superior to HD‐WL in detecting SSLs. In fact, numerically, NBI had a slightly lower SSLDR (7.5%) compared to with HD‐WL (8.0%). This lower detection was occurred despite a longer withdrawal time. Comparing to other NBI studies, Rex et al. reported an increased number of serrated lesions proximal to the sigmoid colon per patient with NBI, however, the results were not statistically significant.^15^ Another multicenter study similarly found no significant improvement in SSLDR using NBI compared to HD‐WL endoscopy in patients with sessile serrated polyposis syndrome.^29^ Additionally, a randomized controlled trial with tandem colonoscopy in patients with previous SSLs also reported no benefit of NBI over HD‐WL.^30^ In a multicenter study with 138 patients, NBI has also failed to improve detection of remnant SSL tissue after EMR of serrated lesions.^31^

Despite NBI not being beneficial in detecting SSL, another key area of potential benefit is in adenoma detection. There appeared to be no statistically significant difference between the two groups in terms of ADR and PDR, however, the odds ratios for these outcomes appeared to favor NBI over HD‐WL (OR: 1.140 for ADR and 1.332 for PDR). It should be noted that ADR and PDR were not our primary study outcomes and our samples size calculations were specifically for SSLDR. After adjusting for various confounding factors, multivariate analysis still showed that NBI use versus HD‐WL use does not significantly improve the PDR or ADR. Results from previous studies have demonstrated that NBI seemed to be better than standard‐definition WL but only equal to HD‐WL with regards to PDR and ADR.^32^, ^33^, ^34^ However, recent studies using the second generation of NBI system which provides brighter illumination showed improved ADR compared to the HD‐WL.^15^, ^35^, ^36^, ^37^ This was also reported in a recent meta‐analysis which found that second‐generation NBI had a better ADR than HD‐WL (OR 1.28; 95% CI, 1.05‐1.56, *p* = 0.02), whereas first‐generation NBI did not.^38^ Although these studies (and ours) have yielded a general trend supporting the use of second generation NBI over HD‐WL, not all results and findings are consistent and more data are needed from future studies.

Our data are consistent with most reported studies for key predictors of polyp and adenoma detection. First, we showed that age was a significant predictor for both polyp detection (OR of 1.115 per year increase) and adenoma detection (OR 1.033 per year increase) on multivariable analysis. The impact of age on PDR and ADR has been demonstrated previously.^39^, ^40^, ^41^ Second, we also confirmed the association between NBI use and increased withdrawal time. Similar results of longer withdrawal time in the NBI arm were noted in previous randomized controlled trials and meta‐analyses.^36^, ^42^, ^43^ It is well‐established that increased withdrawal time leads to improved PDR (as in this study) and ADR.^37^ Since the withdrawal time in the NBI group was longer compared to HD‐WL, one would have expected that the NBI arm should have performed better than HD‐WL in terms of ADR and PDR. Although this was the case numerically in our study, it failed to reach statistical significance.

One hypothesis as to why NBI did not perform better is related to the quality of the bowel preparation. The bowel preparation quality remains key factor in maximizing the benefits of NBI. With NBI, stool appears a reddish color and even a thin film of stool or mucus can significantly impair mucosal visualization and thus, PDR and ADR. This has been demonstrated in a recent meta‐analysis which found the superiority of NBI over HD‐WL most pronounced when the bowel preparation was described as “best” (OR = 1.3; 95% CI, 1.04–1.62; *p* = 0.02) compared to when it was considered only as “adequate” (OR = 1.07; 95% CI, 0.92‐1.24; *p* = 0.38).^38^ However, the overall bowel preparation quality of our subjects was adequate (8 out of 9 on the BBPS) with no significant differences between the two groups which should not have influenced our results substantially.

The major strength of this study is that it was a randomized controlled trial which achieved good homogeneity between the two study arms for most variables. Second, our study had broad inclusion criteria and included a wide variety of ages and indications for colonoscopy. Thus, our findings could be generalizable to the broad population of patients for whom a colonoscopy is indicated. Another strength is that the study used second‐generation NBI and HD‐WL, both of which are the latest, most advanced tools available. This allows for a comparison to be made between two optimal choices rather than between tools which have widely acknowledged shortcomings. Nonetheless, several limitations from this study should be acknowledged. At time of study design, there were few studies reporting SSLDR using NBI to guide us on power calculations. Thus, the cut‐offs we chose were exploratory and derived from studies of SSLDR using HD‐WL and it is possible that the effect size we chose for NBI was too large. Another limitation of the study was the lack of blinding of the colonoscopists to the modalities being investigated. Blinding would be difficult to achieve due to it being immediately obvious to the colonoscopist which modality was being used. Additionally, we did not measure whether there was variation in the SSLDR, PDR, and ADR between operators and over time. Other similar trials have found significant differences between experienced operators. An earlier study found that one operator had a significantly enhanced ADR using NBI while two others did not.^43^ Thus, operators could also have had different baseline ADRs, and adapted to using NBI differently over time. Moreover, patients were randomized to each modality in a 1:1 ratio in blocks of 10. Potentially age, sex, and institution may have influenced the prevalence of lesions. Stratified randomization could have minimized type I error; however, this is especially the case for small trials (<400 patients). Another limitation might be the diagnosis of SSL by a single pathologist. In the past some studies reported interobserver variation among pathologists in the identification of SSLs.^26^

## CONCLUSIONS

In the hands of experienced colonoscopists, NBI does not improve SSLDR compared to HD‐WL. Withdrawal time and patient age remain important factors for polyp and adenoma detection.

## CONFLICT OF INTEREST

The authors declare that they have no conflict of interest.

## FUNDING INFORMATION

None.
